# Concerns about instrumental variable selection for biological effect versus uptake of proton pump inhibitors in Mendelian randomization analysis

**DOI:** 10.1136/gutjnl-2024-332280

**Published:** 2024-12-10

**Authors:** Shuai Yuan, Susanna C. Larsson, Dipender Gill, Stephen Burgess

**Affiliations:** 1 Institute of Environmental Medicine, Karolinska Institutet, Stockholm, Sweden; 2 Department of Surgical Sciences, Uppsala University, Uppsala, Sweden; 3 Department of Epidemiology and Biostatistics, School of Public Health, Imperial College London, London, United Kingdom; 4 MRC Biostatistics Unit, University of Cambridge, Cambridge, UK; 5 Department of Public Health and Primary Care, University of Cambridge, Cambridge, UK

A recent population-based cohort study found an association between proton pump inhibitor (PPI) prescription and risk of inflammatory bowel disease (IBD) which they attributed to protopathic bias (i.e., the drug was prescribed in response to initial disease symptoms) rather than a causal effect of PPI usage [1]. In a following Mendelian randomization (MR) study, An et al selected genetic variants associated with PPI usage to proxy the effect of exposure to PPI treatment [2]. However, interpretation of these results remains unclear due to concerns whether the strategy for instrumental variable selection identifies genetic variants that mimic the treatment under investigation. The fundamental concern is the difference between genetic predictors of the drug target effect versus genetic predictors of taking the drug.

The major concern is that the association between genetic variants and drug use likely stems from their potential to exacerbate the underlying condition targeted by the drug or to intensify associated symptoms. For example, the variant rs11591147 in the *PCSK9* gene region is associated with increased use of cholesterol-lowering medication, but also higher levels of cholesterol [3]. We also found contradictory results between genetically-predicted medication usage behavior and genetically-predicted drug target effect in MR analyses. For example, calcium channel blockers (CCB) have been found to lower risk of stroke and possibly CAD in randomized controlled trials [4]. In Gill *et al*, CCB genetically-proxied by variants mimicking the drug target effect (variants in genes encoding calcium channel subunits) was associated with lower risk of stroke and CAD [5]. We here conducted an MR analysis to examine associations of genetically-predicted CCB usage with stroke and CAD. Genetic variants associated with CCB usage were extracted from a genome-wide meta-analysis of UK Biobank and FinnGen studies [6]. Summary-level data for systolic blood pressure (SBP), stroke, and CAD were obtained from large-scale genome-wide meta-analyses [7, 8, 9]. More information on this MR analysis is presented in online supplements. After data harmonization, we compared variant-phenotype associations and found many variant associations with increased CCB usage corresponded with higher SBP and higher risk of stroke and CAD (Figure 1A). This is contrary to the known SBP lowering effect of the drug. MR analysis further observed that genetic-predicted CCB usage was associated with increased risk of stroke and CAD (Figure 1B and 1C and online supplements). Again, this is contrary to what has been seen in trials.

There are several reasons for these discrepancies. First, even if these variants heighten the likelihood of PPI use, individuals may have spent a significant duration of their lives without prescription intervention. Second, identifying specific genetic predictors of PPI uptake is challenging, as drug prediction is presumed to be influenced by social and behavioral determinants. Thus, the used genetic instruments are more likely to predict medication behaviors largely influenced by social and behavioral factors instead of the biological effects of PPIs, and hence are likely to be pleiotropic. Even in the hypothetical scenario of identifying specific genetic predictors associated with PPI consumption, the interpretational clarity of findings remains uncertain. These concerns also apply to MR studies using behavioral proxies of exposures. For example, genetic predictors of smoking behavior could be misleading if used to understand the effects of elevated nicotine levels.

In summary, drug target MR analyses should focus on finding genetic variants that perturb the drug target [10]. This rule also applies to MR analyses for other behaviors where the genetic variants leveraged as instruments should ideally be biologically relevant to the exposure mechanism, not simply genetic predictors of greater exposure levels. Thus, the strategy of using genetic predictors of drug prescription or drug usage is not recommended.

## Supplementary Material

Supplementary material

## Figures and Tables

**Figure 1:**
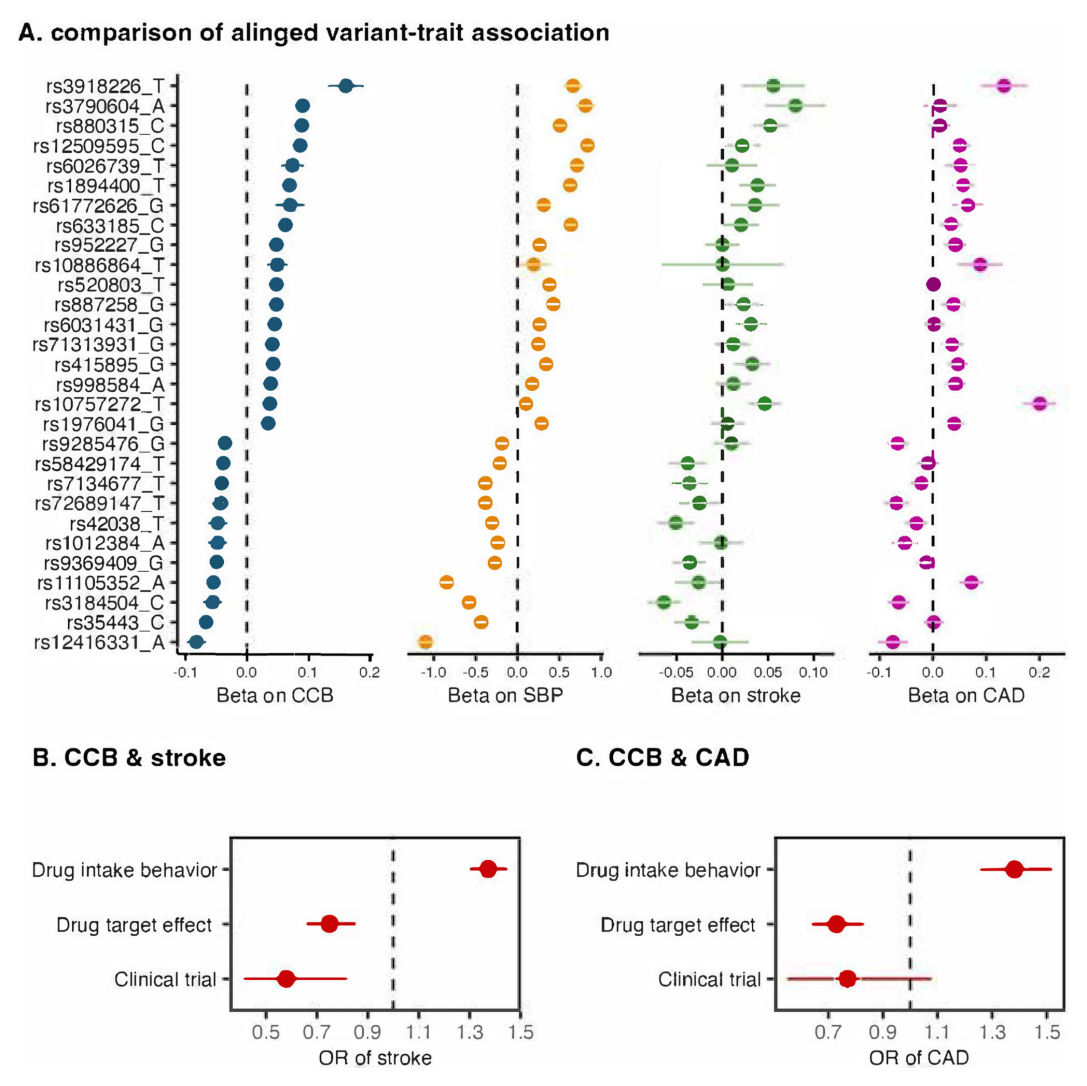
Genetic associations with calcium channel blocker (CCB) usage and known effects of CCBs. Plot A: Among genetic predictors of CCB usage, 29 variants had associations with increased CCB usage, higher systolic blood pressure (SBP), and higher risk of stroke and coronary artery disease (CAD). The y axis labels represent rsID and effect allele. Plots B and C compare estimates for the effect of CCB usage on risk of stroke and CAD from: i) MR analysis, where CCB was proxied by genetic predictors of CCB usage, ii) MR analysis, where CCB was proxied by variants that mimic the drug target effect, and iii) clinical trials. The estimates in clinical trials were obtained from Wright JM, Musini VM, Gill R. First-line drugs for hypertension. Cochrane Database Syst Rev. 2018; 4:CD001841. Abbreviation: OR, odds ratio.
