# Supplementary material for: Concerns about instrumental variable selection for biological effect versus uptake of proton pump inhibitors in Mendelian randomization analysis
Source: Gut. Author manuscript; Available in PMC 2024 Nov 18. (PMC7616827; doi:10.1136/gutjnl-2024-332280)
Supplement: Supplementary material [file EMS197947-supplement-Supplementary_material.docx]

Supplements for

**Concerns about instrumental variable selection for biological effect versus uptake of proton pump inhibitors in Mendelian randomization analysis**

Shuai Yuan, Susanna C. Larsson, Dipender Gill, Stephen Burgess

**Genetic instrument selection**

Single nucleotide polymorphisms (SNPs) associated with calcium channel blocker usage at the genome-wide significance level (*P* <5×10^-8^) were obtained from a genome-wide meta-analysis of UK Biobank and FinnGen studies [1]. Among these SNPs, we estimated the genetic correlation matrix based on the 1000 Genomes European panel and selected independent SNPs with *R*^2^ <0.001 as the instrumental variables. We calculated the *F* statistic using an approximation method (β^2^/standard error^2^) for each SNP and found included SNPs with higher *F* statistic (*F_minimum_*=29.4 and *F_average_*=55.2) >10, which suggested that weak instrument bias is less likely. To minimize horizontal pleiotropy, we removed SNPs near *FTO* (rs56094641) and *APOE* (rs7412) gene with potential pleiotropic effects, leaving 107 SNPs used as instrumental variables. Detailed information on SNPs is presented in **supplementary table 1**.

**Outcome data source**

Summary-level data for systolic blood pressure (SBP), stroke, and coronary artery disease (CAD) were obtained from large-scale genome-wide meta-analyses [2, 3, 4]. Genome-wide meta-analysis of SNP included 757,601 individuals of European ancestry [2]. Genome-wide meta-analysis on stroke [3] and CAD [4] included 40,585 stroke cases and 406,111 controls and 60,801 CAD cases and 123,504 controls, respectively. Detailed information on phenotype definition, quality control, and adjustment, refers to the original studies [2, 3, 4].

**Statistical analysis**

Data were harmonized by effect and non-effect alleles. Eight palindromic SNPs with minor allele frequency >0.4 were removed from the analysis. The inverse variance weighted method under multiplicative random effects was used as the primary analysis. Since the primary analysis is sensitive to SNP outliers, we conducted three sensitivity analyses, including the weighted median [5], MR-Egger [6], and MR-PRESSO [7] to examine the robustness of the results and detect and adjust for horizontal pleiotropy. The weighted median method provides consistent estimates when more than half of weight comes from valid SNPs [5]. MR-Egger can detect pleiotropy by its intercept test and provide estimate after adjustment for pleiotropy; however, its estimates are usually underpowered [6]. MR-PRESSO can identify outlying SNPs and generate estimates after the removal of outlier SNPs [7]. Cochran’s Q value was estimated to assess the heterogeneity. The MR association with a *P* value <0.05 was deemed statistically significant. All tests were two sided and done using the TwoSampleMR [8] package in the R software (version 4.0.2).

**Results**

Genetically predicted calcium channel blocker usage was associated with increased levels of SBP and increased risk of stroke and CAD (**supplementary table 2**). The associations were consistent in sensitivity analyses (**supplementary table 2**). High heterogeneity was observed in these associations; however, no indication of horizontal pleiotropy was detected by MR-Egger intercept test (*P*>0.05). MR-PRESSO identified a few SNP outliers. But the associations remained stable after the removal of identified outlying SNPs.

**References:**

1 Sakaue S, Kanai M, Tanigawa Y, Karjalainen J, Kurki M, Koshiba S*, et al.* A cross-population atlas of genetic associations for 220 human phenotypes. Nat Genet 2021;**53**:1415-24.

2 Evangelou E, Warren HR, Mosen-Ansorena D, Mifsud B, Pazoki R, Gao H*, et al.* Genetic analysis of over 1 million people identifies 535 new loci associated with blood pressure traits. Nat Genet 2018;**50**:1412-25.

3 Malik R, Chauhan G, Traylor M, Sargurupremraj M, Okada Y, Mishra A*, et al.* Multiancestry genome-wide association study of 520,000 subjects identifies 32 loci associated with stroke and stroke subtypes. Nat Genet 2018;**50**:524-37.

4 Nikpay M, Goel A, Won HH, Hall LM, Willenborg C, Kanoni S*, et al.* A comprehensive 1,000 Genomes-based genome-wide association meta-analysis of coronary artery disease. Nat Genet 2015;**47**:1121-30.

5 Burgess S, Bowden J, Fall T, Ingelsson E, Thompson SG. Sensitivity analyses for robust causal inference from Mendelian randomization analyses with multiple genetic variants. Epidemiology 2017;**28**:30-42.

6 Burgess S, Thompson SG. Interpreting findings from Mendelian randomization using the MR-Egger method. European journal of epidemiology 2017;**32**:377-89.

7 Verbanck M, Chen CY, Neale B, Do R. Detection of widespread horizontal pleiotropy in causal relationships inferred from Mendelian randomization between complex traits and diseases. Nature genetics 2018;**50**:693-8.

8 Hemani G, Zheng J, Elsworth B, Wade KH, Haberland V, Baird D*, et al.* The MR-Base platform supports systematic causal inference across the human phenome. Elife 2018;**7**.

**Supplementary table 1**. Genetc instruments to proxy calcium channel blocker usage

| **SNP** | **chr** | **position** | **Nearby genes** | **EA** | **NEA** | **EAF** | **Beta** | **SE** | ***P* value** | ***F* statistic** | **Include** |
| --- | --- | --- | --- | --- | --- | --- | --- | --- | --- | --- | --- |
| rs3790604 | 1 | 113046879 | *WNT2B* | A | C | 0.18 | 0.093 | 0.008 | 1.10E-32 | 142.5 | YES |
| rs4971099 | 1 | 155155608 | *TRIM46* | G | A | 0.82 | -0.071 | 0.011 | 3.44E-11 | 44.2 | YES |
| rs57748895 | 1 | 115826169 | *-* | T | A | 0.02 | 0.175 | 0.031 | 9.02E-09 | 33 | YES |
| rs59180873 | 1 | 27848634 | *-* | G | A | 0.08 | -0.083 | 0.015 | 3.58E-08 | 30.3 | YES |
| rs61772626 | 1 | 57015668 | *PLPP3* | G | A | 0.07 | 0.071 | 0.012 | 7.02E-09 | 33.4 | YES |
| rs880315 | 1 | 10796866 | *CASZ1* | C | T | 0.50 | 0.088 | 0.006 | 4.72E-47 | 209.5 | YES |
| rs12473088 | 2 | 162485073 | *SLC4A10* | G | A | 0.10 | -0.054 | 0.01 | 3.41E-08 | 30.4 | YES |
| rs1275988 | 2 | 26914364 | *KCNK3* | T | C | 0.43 | -0.085 | 0.006 | 2.15E-41 | 183.3 | YES |
| rs4672441 | 2 | 61638952 | *USP34* | C | T | 0.66 | 0.036 | 0.006 | 1.97E-09 | 36.4 | YES |
| rs73029563 | 2 | 165008166 | *-* | G | C | 0.56 | 0.046 | 0.006 | 2.74E-15 | 62.4 | NO |
| rs952227 | 2 | 227062080 | *-* | G | A | 0.78 | 0.05 | 0.007 | 4.62E-12 | 48 | YES |
| rs12638862 | 3 | 169477506 | *-* | G | A | 0.44 | -0.035 | 0.006 | 1.54E-08 | 32.4 | YES |
| rs1290786 | 3 | 169097381 | *MECOM* | T | C | 0.29 | 0.046 | 0.007 | 9.14E-12 | 46.7 | YES |
| rs35593046 | 3 | 53553923 | *CACNA1D* | T | G | 0.40 | -0.058 | 0.006 | 6.52E-21 | 87.8 | YES |
| rs3889027 | 3 | 168836505 | *MECOM* | T | G | 0.08 | -0.068 | 0.011 | 1.63E-10 | 41.3 | YES |
| rs4605535 | 3 | 53602285 | *CACNA1D* | T | C | 0.33 | 0.045 | 0.006 | 3.86E-13 | 53.1 | YES |
| rs648103 | 3 | 27550301 | *-* | C | T | 0.74 | -0.067 | 0.007 | 2.37E-22 | 95.6 | YES |
| rs9854769 | 3 | 185520948 | *IGF2BP2* | G | A | 0.33 | 0.035 | 0.006 | 1.23E-08 | 32.5 | YES |
| rs10015412 | 4 | 144162582 | *-* | C | A | 0.74 | -0.041 | 0.007 | 2.05E-09 | 36.4 | YES |
| rs11726072 | 4 | 187543527 | *-* | A | G | 0.12 | -0.068 | 0.012 | 3.40E-08 | 30.5 | YES |
| rs12509595 | 4 | 81182554 | *-* | C | T | 0.29 | 0.086 | 0.006 | 1.28E-42 | 186.3 | YES |
| rs1976041 | 4 | 156486040 | *-* | G | A | 0.64 | 0.034 | 0.006 | 4.70E-08 | 29.7 | YES |
| rs6848906 | 4 | 111386198 | *ENPEP* | C | T | 0.73 | -0.05 | 0.007 | 8.15E-14 | 56.6 | YES |
| rs72689147 | 4 | 156639888 | *GUCY1A1* | T | G | 0.21 | -0.043 | 0.007 | 1.04E-09 | 36.9 | YES |
| rs73249870 | 4 | 26809019 | *-* | T | C | 0.26 | -0.042 | 0.007 | 9.11E-10 | 37.8 | YES |
| rs10054208 | 5 | 55688992 | *-* | T | C | 0.45 | 0.035 | 0.006 | 4.49E-09 | 34.6 | YES |
| rs10061288 | 5 | 114389826 | *-* | G | A | 0.55 | -0.04 | 0.006 | 5.46E-12 | 47.1 | YES |
| rs1694068 | 5 | 53283630 | *ARL15* | A | T | 0.71 | 0.036 | 0.007 | 2.87E-08 | 31 | YES |
| rs2962383 | 5 | 157494859 | *-* | T | C | 0.18 | 0.047 | 0.008 | 2.32E-09 | 36 | YES |
| rs6898449 | 5 | 127846045 | *FBN2* | G | A | 0.35 | 0.049 | 0.006 | 9.94E-16 | 64.3 | YES |
| rs7701003 | 5 | 157824481 | *LINC02227* | G | A | 0.37 | -0.06 | 0.008 | 5.35E-13 | 52.1 | YES |
| rs7733331 | 5 | 32828846 | *-* | C | T | 0.59 | 0.043 | 0.006 | 2.39E-13 | 54.2 | YES |
| rs1552886 | 6 | 151019750 | *PLEKHG1* | G | A | 0.96 | 0.086 | 0.014 | 8.85E-10 | 37.5 | YES |
| rs35932914 | 6 | 32622150 | *-* | A | G | 0.08 | 0.092 | 0.016 | 4.70E-09 | 34.3 | YES |
| rs520803 | 6 | 32188603 | *NOTCH4* | T | C | 0.26 | 0.048 | 0.007 | 1.80E-13 | 53.6 | YES |
| rs72836474 | 6 | 26356451 | *-* | T | C | 0.02 | 0.138 | 0.021 | 5.60E-11 | 42.9 | YES |
| rs9285476 | 6 | 134159976 | *TARID* | G | C | 0.38 | -0.035 | 0.006 | 3.91E-09 | 34.8 | YES |
| rs9369409 | 6 | 43346462 | *-* | G | C | 0.62 | -0.05 | 0.006 | 4.48E-17 | 70.4 | YES |
| rs9375461 | 6 | 127159310 | *-* | A | C | 0.44 | 0.064 | 0.006 | 1.97E-28 | 122.9 | YES |
| rs998584 | 6 | 43757896 | *VEGFA* | A | C | 0.51 | 0.038 | 0.006 | 2.20E-11 | 45.4 | YES |
| rs10254101 | 7 | 151415536 | *PRKAG2* | T | C | 0.17 | 0.049 | 0.008 | 1.74E-09 | 36.3 | YES |
| rs12705389 | 7 | 106405642 | *-* | T | C | 0.18 | 0.054 | 0.008 | 2.22E-12 | 48.6 | YES |
| rs13234269 | 7 | 130429186 | *-* | A | T | 0.41 | -0.04 | 0.006 | 6.58E-11 | 43.3 | NO |
| rs143524414 | 7 | 98965615 | *ARPC1A* | A | G | 0.04 | -0.083 | 0.015 | 1.67E-08 | 31.9 | YES |
| rs3918226 | 7 | 150690176 | *NOS3* | T | C | 0.08 | 0.161 | 0.015 | 4.12E-26 | 111.1 | YES |
| rs42038 | 7 | 92243719 | *CDK6* | T | C | 0.17 | -0.047 | 0.008 | 2.10E-08 | 31.7 | YES |
| rs4722675 | 7 | 27243962 | *-* | G | A | 0.78 | 0.071 | 0.007 | 9.57E-22 | 91.3 | YES |
| rs6961048 | 7 | 27328187 | *-* | G | C | 0.08 | 0.086 | 0.011 | 8.99E-15 | 60.6 | YES |
| rs7811577 | 7 | 1102829 | *C7orf50* | G | C | 0.48 | 0.034 | 0.006 | 2.26E-09 | 36.2 | NO |
| rs782507 | 7 | 134203428 | *-* | T | C | 0.48 | -0.041 | 0.006 | 6.79E-13 | 52.5 | YES |
| rs13273172 | 8 | 11461111 | *-* | G | A | 0.47 | -0.047 | 0.008 | 4.17E-09 | 34.2 | YES |
| rs2977324 | 8 | 76716737 | *-* | G | T | 0.65 | 0.045 | 0.006 | 1.60E-13 | 53.9 | YES |
| rs35783704 | 8 | 105966258 | *ZFPM2* | A | G | 0.05 | -0.081 | 0.014 | 2.11E-09 | 35.7 | YES |
| rs58429174 | 8 | 26011922 | *-* | T | C | 0.26 | -0.037 | 0.007 | 1.40E-08 | 32.6 | YES |
| rs7463212 | 8 | 143991858 | *GML* | A | T | 0.45 | -0.052 | 0.006 | 4.81E-19 | 78.9 | NO |
| rs7815731 | 8 | 95746833 | *DPY19L4* | G | C | 0.50 | 0.033 | 0.006 | 4.62E-08 | 29.4 | NO |
| rs10757272 | 9 | 22088260 | *CDKN2B-AS1* | T | C | 0.56 | 0.037 | 0.006 | 2.54E-10 | 39.3 | YES |
| rs10993958 | 9 | 136535677 | *SARDH* | A | G | 0.07 | -0.091 | 0.016 | 6.46E-09 | 33.8 | YES |
| rs1250505 | 9 | 128193456 | *-* | T | G | 0.38 | -0.041 | 0.006 | 1.12E-11 | 45.8 | YES |
| rs28558845 | 9 | 4334791 | *GLIS3* | C | G | 0.21 | -0.041 | 0.007 | 9.21E-09 | 33.3 | YES |
| rs76038906 | 9 | 113250200 | *SVEP1* | T | G | 0.03 | 0.123 | 0.022 | 2.20E-08 | 31.5 | YES |
| rs10886864 | 10 | 122929537 | *-* | T | C | 0.85 | 0.05 | 0.009 | 5.58E-09 | 33.9 | YES |
| rs11187838 | 10 | 96038686 | *PLCE1* | A | G | 0.44 | -0.045 | 0.006 | 6.11E-15 | 60.2 | YES |
| rs117464403 | 10 | 107158054 | *-* | A | G | 0.02 | 0.182 | 0.029 | 5.38E-10 | 38.5 | YES |
| rs12416331 | 10 | 104928914 | *-* | A | T | 0.16 | -0.083 | 0.008 | 3.76E-25 | 108.2 | YES |
| rs1277748 | 10 | 18520535 | *CACNB2* | A | G | 0.55 | -0.033 | 0.006 | 2.64E-08 | 31 | YES |
| rs1658425 | 10 | 60331547 | *BICC1* | C | G | 0.51 | 0.043 | 0.006 | 5.50E-14 | 57.4 | NO |
| rs60457246 | 10 | 63433490 | *-* | A | G | 0.16 | -0.095 | 0.011 | 1.01E-17 | 73.4 | YES |
| rs7070847 | 10 | 18726054 | *CACNB2* | A | G | 0.16 | -0.058 | 0.009 | 3.09E-11 | 44.1 | YES |
| rs74157561 | 10 | 115725280 | *-* | G | A | 0.09 | 0.059 | 0.01 | 2.42E-09 | 36 | YES |
| rs10835920 | 11 | 32489664 | *WT1-AS* | T | C | 0.53 | 0.034 | 0.006 | 2.82E-08 | 30.7 | YES |
| rs415895 | 11 | 9769562 | *SWAP70* | G | C | 0.52 | 0.042 | 0.006 | 1.18E-12 | 50.9 | NO |
| rs525028 | 11 | 116705516 | *APOC3* | A | G | 0.49 | -0.045 | 0.007 | 8.93E-12 | 47.1 | YES |
| rs562434 | 11 | 1892930 | *LSP1* | A | G | 0.35 | 0.096 | 0.008 | 5.33E-30 | 129.3 | YES |
| rs633185 | 11 | 100593538 | *ARHGAP42* | C | G | 0.59 | 0.062 | 0.006 | 1.26E-24 | 106.4 | YES |
| rs75162774 | 11 | 65492483 | *RNASEH2C* | A | G | 0.24 | 0.043 | 0.007 | 5.87E-10 | 38.3 | YES |
| rs751984 | 11 | 61278246 | *-* | C | T | 0.31 | -0.047 | 0.007 | 9.85E-12 | 46.2 | YES |
| rs11105352 | 12 | 90026462 | *ATP2B1* | A | G | 0.26 | -0.053 | 0.007 | 1.03E-15 | 63.5 | YES |
| rs2024077 | 12 | 2450595 | *CACNA1C* | A | G | 0.61 | 0.036 | 0.006 | 1.28E-09 | 37.2 | YES |
| rs2024385 | 12 | 12888438 | *APOLD1* | A | T | 0.46 | -0.037 | 0.006 | 1.71E-10 | 40.9 | NO |
| rs2377585 | 12 | 8932201 | *RIMKLB* | G | T | 0.27 | -0.056 | 0.009 | 3.97E-10 | 39 | YES |
| rs3184504 | 12 | 111884608 | *SH2B3* | C | T | 0.74 | -0.056 | 0.008 | 2.22E-12 | 49.5 | YES |
| rs35443 | 12 | 115552878 | *-* | C | G | 0.32 | -0.067 | 0.006 | 5.06E-27 | 115.4 | YES |
| rs7134677 | 12 | 54441498 | *HOXC4* | T | C | 0.35 | -0.041 | 0.006 | 2.02E-11 | 45 | YES |
| rs277136 | 13 | 32210101 | *-* | A | G | 0.49 | 0.075 | 0.008 | 5.99E-21 | 88.6 | YES |
| rs682709 | 13 | 22294082 | *-* | G | C | 0.82 | 0.063 | 0.009 | 3.03E-13 | 52.9 | YES |
| rs28415454 | 14 | 98616922 | *LINC02295* | T | C | 0.20 | 0.057 | 0.01 | 1.14E-08 | 32.6 | YES |
| rs12906962 | 15 | 95312071 | *-* | C | T | 0.29 | 0.036 | 0.006 | 2.39E-08 | 31.5 | YES |
| rs1378941 | 15 | 75080150 | *CSK* | A | C | 0.45 | -0.047 | 0.007 | 6.44E-13 | 51.4 | YES |
| rs1894400 | 15 | 91428955 | *FES* | T | C | 0.22 | 0.072 | 0.007 | 4.04E-22 | 93.6 | YES |
| rs4778848 | 15 | 81019572 | *ABHD17C* | A | C | 0.28 | 0.065 | 0.007 | 5.33E-19 | 80.3 | YES |
| rs467357 | 16 | 89717397 | *CHMP1A* | G | A | 0.23 | -0.065 | 0.008 | 2.36E-16 | 67.3 | YES |
| rs56094641 | 16 | 53806453 | *FTO* | G | A | 0.31 | 0.052 | 0.006 | 1.83E-16 | 67 | NO |
| rs77924615 | 16 | 20392332 | *PDILT* | A | G | 0.20 | -0.04 | 0.007 | 2.63E-08 | 30.7 | YES |
| rs9888752 | 16 | 81590971 | *-* | T | C | 0.41 | -0.048 | 0.008 | 6.29E-09 | 33.3 | YES |
| rs9922008 | 16 | 75335406 | *CFDP1* | C | T | 0.61 | 0.036 | 0.006 | 9.61E-10 | 37.6 | YES |
| rs1012384 | 17 | 59196064 | *BCAS3* | A | G | 0.83 | -0.048 | 0.008 | 6.65E-10 | 37.7 | YES |
| rs197920 | 17 | 45005095 | *GOSR2* | T | C | 0.34 | 0.036 | 0.006 | 3.03E-09 | 35.6 | YES |
| rs887258 | 17 | 59479580 | *TBX2-AS1* | G | C | 0.73 | 0.048 | 0.007 | 1.36E-13 | 54.5 | YES |
| rs321901 | 18 | 48917638 | *LINC01630* | C | A | 0.33 | 0.048 | 0.009 | 2.80E-08 | 30.6 | YES |
| rs12978472 | 19 | 7257990 | *-* | G | C | 0.13 | -0.109 | 0.012 | 2.60E-19 | 81 | YES |
| rs7412 | 19 | 45412079 | *APOE* | T | C | 0.06 | -0.078 | 0.012 | 9.56E-11 | 41.7 | NO |
| rs1887320 | 20 | 10965998 | *-* | A | G | 0.48 | 0.046 | 0.006 | 6.43E-16 | 66 | YES |
| rs6026739 | 20 | 57739469 | *ZNF831* | T | A | 0.10 | 0.074 | 0.01 | 1.59E-14 | 58.5 | YES |
| rs6031431 | 20 | 42795152 | *-* | G | A | 0.39 | 0.045 | 0.006 | 6.22E-14 | 56.8 | YES |
| rs6108171 | 20 | 8635551 | *PLCB1* | T | A | 0.29 | -0.041 | 0.006 | 2.36E-10 | 40.2 | YES |
| rs8118848 | 20 | 62461572 | *-* | A | G | 0.19 | -0.048 | 0.008 | 1.90E-10 | 40.8 | YES |
| rs8123890 | 20 | 50106713 | *NFATC2* | G | A | 0.43 | 0.036 | 0.006 | 1.11E-09 | 37.5 | YES |
| rs71313931 | 22 | 19960184 | *COMT* | G | C | 0.24 | 0.042 | 0.007 | 4.86E-10 | 38.9 | YES |

EA, effect allele; EAF, effet allele frequency; NEA, non-effect allele; SE, standard error; SNP, single nucleotide polymorphisms.

**Supplementary table 2.** Associations of genetically predicted calcium channel blocker usage with systolic blood pressure, stroke, and coronary artery disease

| **Outcomes** | **nSNPs** | **Cochran’s Q** | ***P_MR-Egger intercept_*** | **Inverse variance weighted** | | | **Weighted median** | | |
| --- | --- | --- | --- | --- | --- | --- | --- | --- | --- |
|  |  |  |  | **Estimate** | **95% CI** | ***P*** | **Estimate** | **95% CI** | ***P*** |
| Systolic blood pressure | 95 | 1818 | 0.326 | 6.48 | 5.92-7.04 | 1.06E-114 | 5.46 | 5.05-5.87 | 1.88E-150 |
| Stroke | 99 | 192 | 0.536 | 1.37 | 1.3-1.45 | 1.32E-30 | 1.35 | 1.27-1.44 | 1.22E-19 |
| Coronary heart disease | 97 | 533 | 0.115 | 1.38 | 1.26-1.52 | 2.13E-11 | 1.25 | 1.16-1.35 | 1.99E-09 |
|  |  |  |  | **MR-Egger** | | | **MR-PRESSO** | | |
|  |  |  |  | **Estimate** | **95% CI** | ***P*** | **Estimate** | **95% CI** | ***P*** |
| Systolic blood pressure | 95 | 1818 | 0.326 | 5.70 | 4.05-7.35 | 1.20E-09 | 6.25 | 5.85-6.65 | 9.46E-46 |
| Stroke | 99 | 192 | 0.536 | 1.31 | 1.12-1.54 | 1.38E-03 | 1.35 | 1.28-1.42 | 1.15E-20 |
| Coronary heart disease | 97 | 533 | 0.115 | 1.11 | 0.84-1.48 | 4.65E-01 | 1.36 | 1.28-1.45 | 2.37E-15 |

The estimate was beta for systolic blood pressure in mmHg and odds ratio for stroke and coronary artery disease. CI, confidence interval; SNPs, single nucleotide polymorphisms. The estimates from MR-PRESSO were estimates after the removal of outliers.
